# Supplementary material for: Exfoliation of natural van der Waals heterostructures to a single unit cell thickness
Source: Nat Commun. 2017 Feb 13;8:14410. doi: 10.1038/ncomms14410 (PMC5316834; doi:10.1038/ncomms14410)
Supplement: Supplementary Information — Supplementary Figures, Supplementary Tables, Supplementary Notes and Supplementary References [file ncomms14410-s1.pdf]

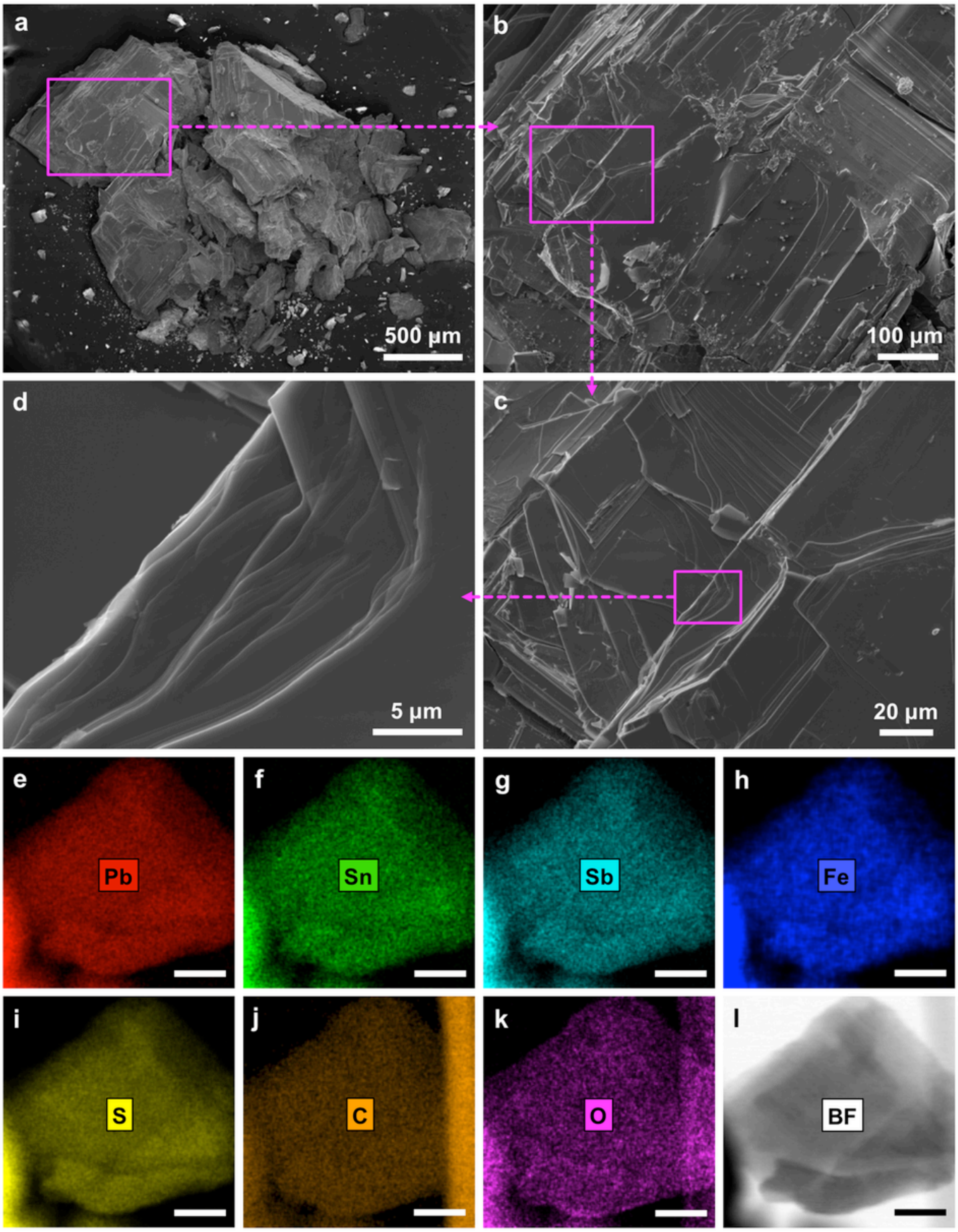

Supplementary Figure 1 | SEM images and TEM-EDXS mapping of franckeite. a–d, SEM images of a franckeite crystal immobilised on a conductive carbon support. Zoom areas are highlighted in (**a**–**c**) by the magenta rectangles. **e**–**k**, EDXS maps of lead, tin, antimony, iron, sulphur, carbon, and oxygen, respectively, within another crystal. **l**, BF-TEM image of the same crystal. The scale bars in (**e**–**l**) denote 30 nm. Electron beam accelerating voltages of 15 kV and 200 kV were used for the SEM and TEM-EDXS, respectively.

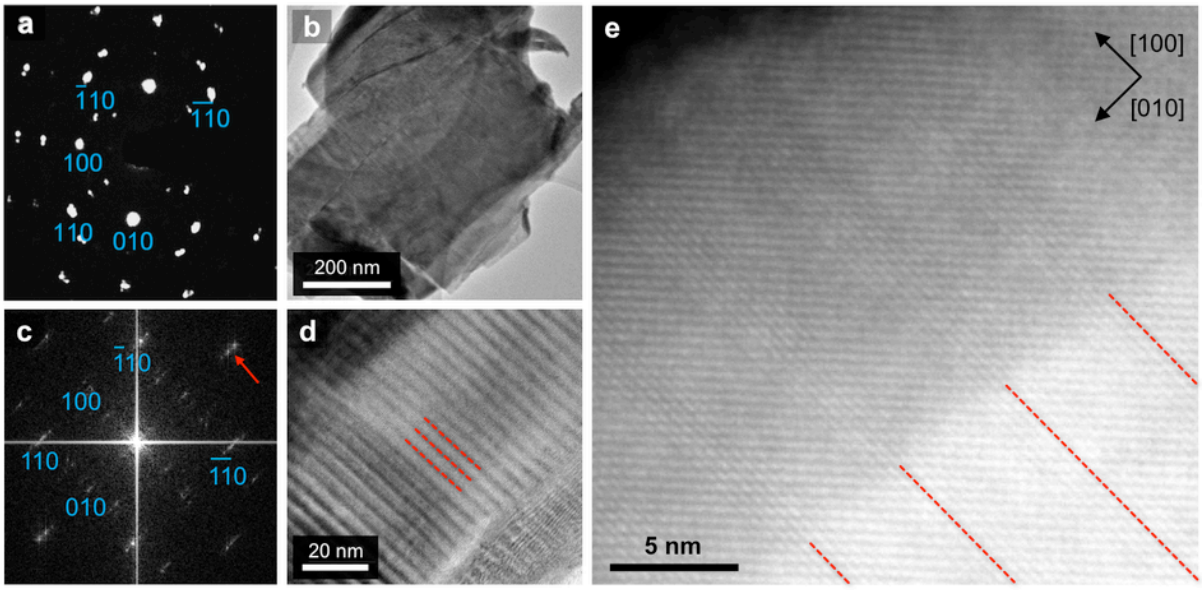

Supplementary Figure 2 | TEM imaging of franckeite viewed along the [001] direction (perpendicular to the basal plane). **a,** Indexed electron diffraction pattern. **b,d,e,** TEM images of franckeite viewed along the [001] direction. **c,** corresponding Fourier transform of the high resolution image in (**e**). These TEM images clearly show the ~4.4 nm moiré fringes, highlighted by the red dashed lines in (**d**) and (**e**). These fringes are also present in the Fourier transform image (**c**), causing additional spots indicated by the red arrow to appear.

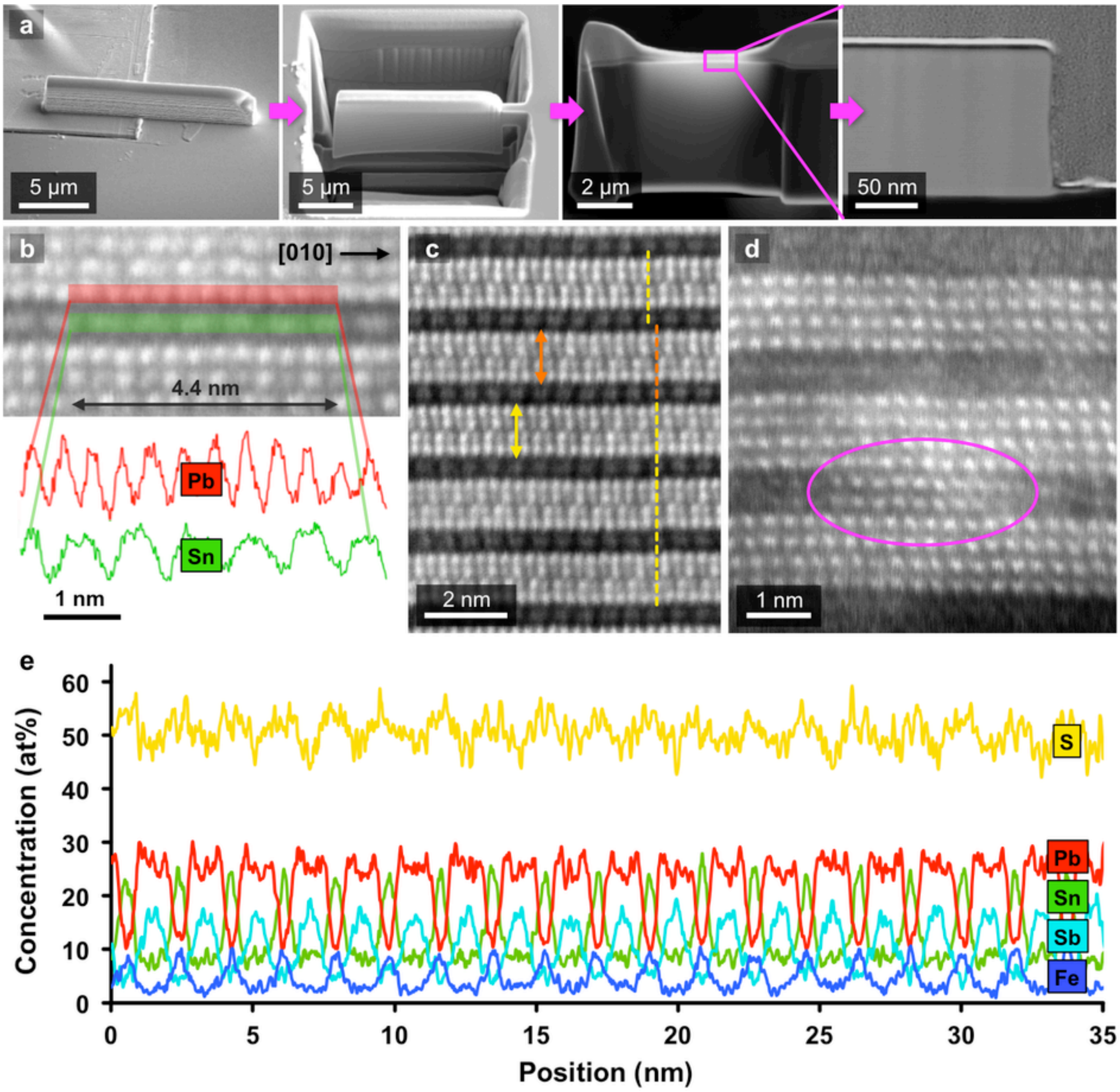

Supplementary Figure 3 | Cross-sectional HAADF-STEM imaging of franckeite along the [010] direction (parallel to the basal plane). **a**, SEM and STEM images illustrating the process of FIB cross-sectional sample preparation from a crystal mechanically exfoliated on an SiO_2/Si substrate. **b**, Intensity line profiles reveal the incommensurate stacking between the H layer (green) and the adjacent T layer (red). **c**, Variations in stacking between the consecutive H layers. The yellow arrow separates the H layers in which the Sn atoms are positioned on top of one another, whereas the orange arrow indicates the H layers that have a relative displacement along the [010] direction. **d**, Electron beam induced restructuring of the H layer, where a continuous PbS link between Pb-rich layers is formed. **e,** EDXS concentration profiles of a large portion of the cross-section.

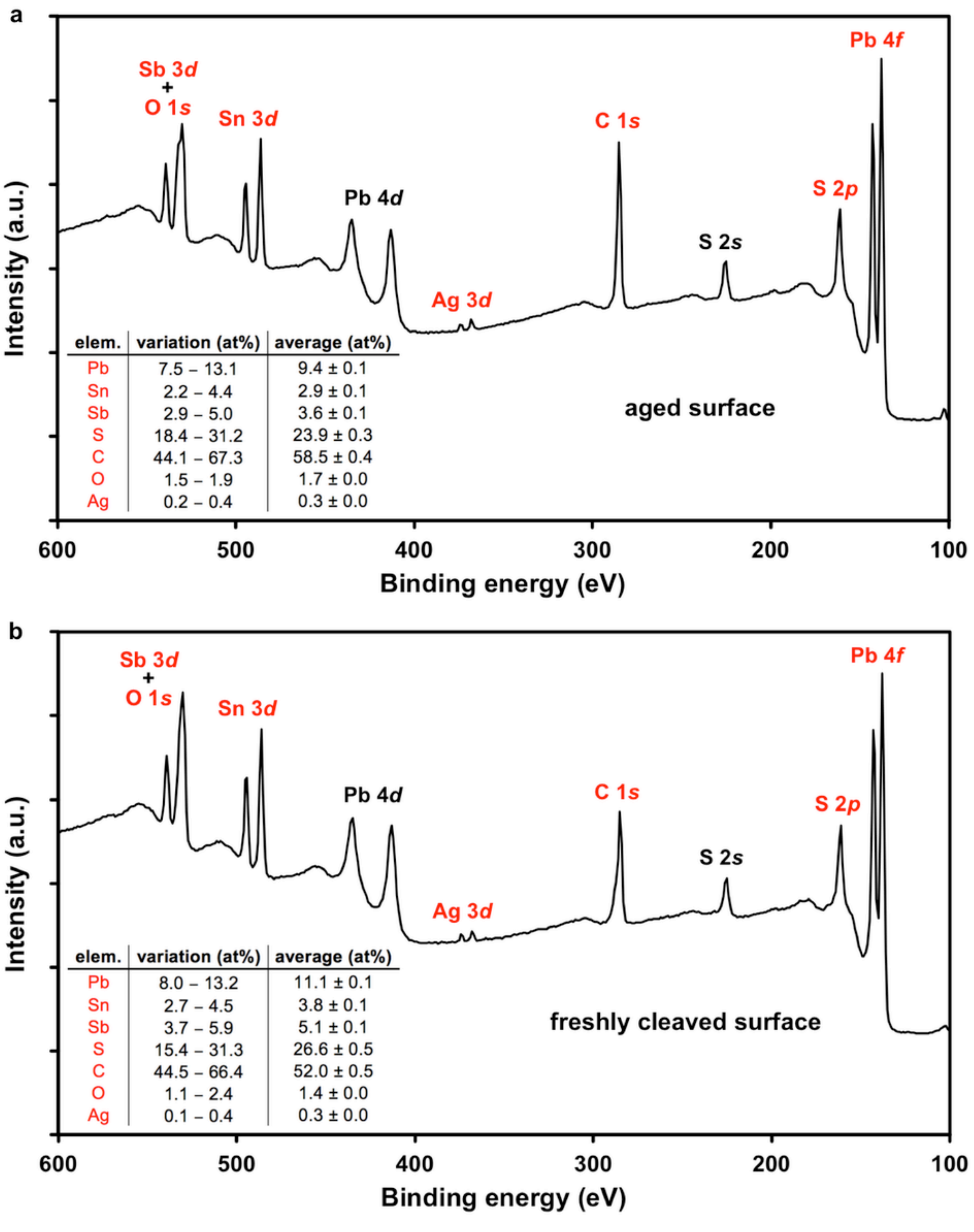

Supplementary Figure 4 | XPS of the aged and freshly cleaved franckeite surface. a, XPS spectrum of the aged franckeite surface. **b**, XPS spectrum of the freshly cleaved franckeite surface. Each spectrum is an average of 5 individual measurements from different parts of the crystal. The peaks used for the quantification in the inset tables are labelled in red, other major peaks are labelled in black. The spectra are normalised to the intensity of the Pb $4f_{7/2}$ peak (137.6 eV) with the adventitious carbon C 1*s* peak positioned at 284.7 eV.[1]

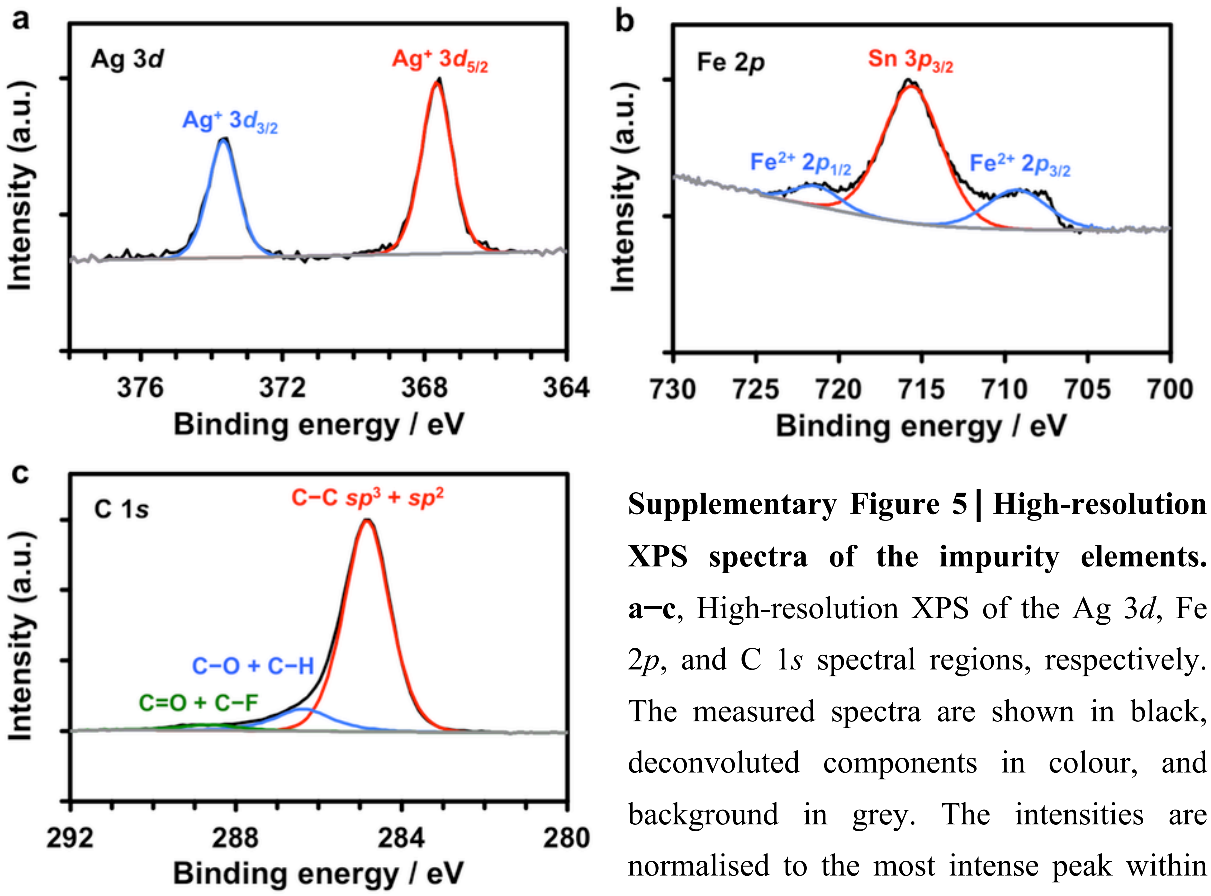

Supplementary Figure 5 | High-resolution XPS spectra of the impurity elements. **a−c**, High-resolution XPS of the Ag 3*d*, Fe 2*p*, and C 1*s* spectral regions, respectively. The measured spectra are shown in black, deconvoluted components in colour, and background in grey. The intensities are normalised to the most intense peak within the respective spectral region.

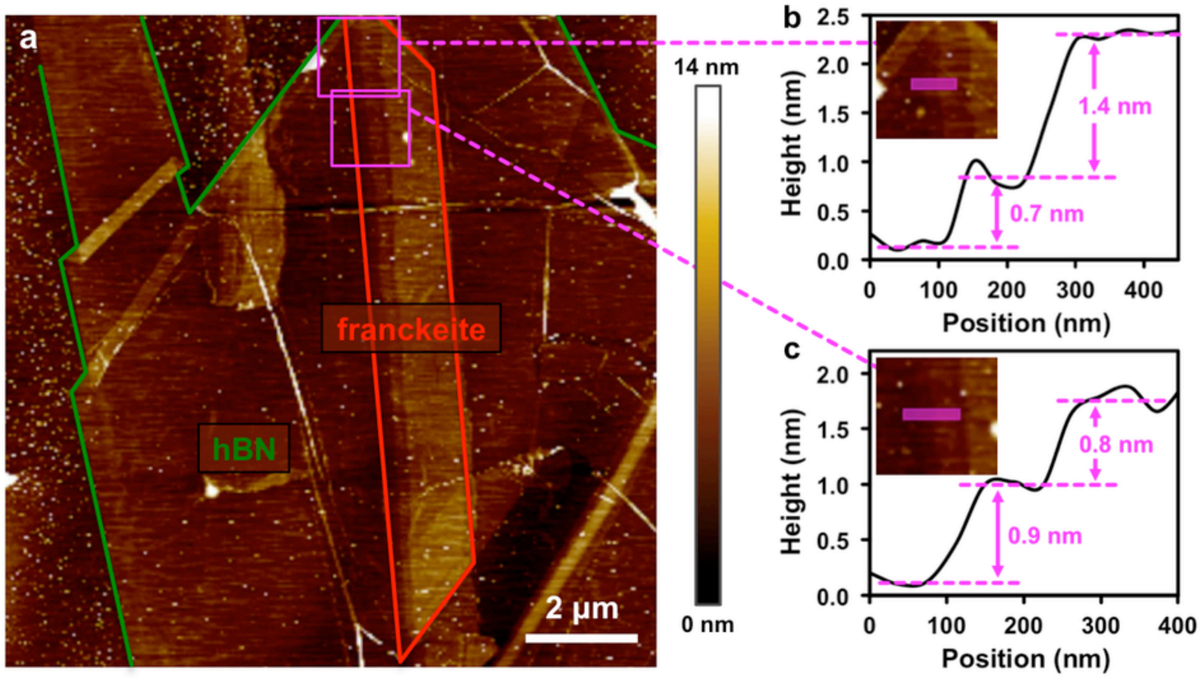

Supplementary Figure 6 | AFM characterisation of franckeite with a sub-monolayer thickness. a, AFM image of a franckeite crystal (red) encapsulated in hBN crystal (green). **b−c,** Height profiles of the sub-monolayer terraces taken from areas indicated by the magenta rectangles.

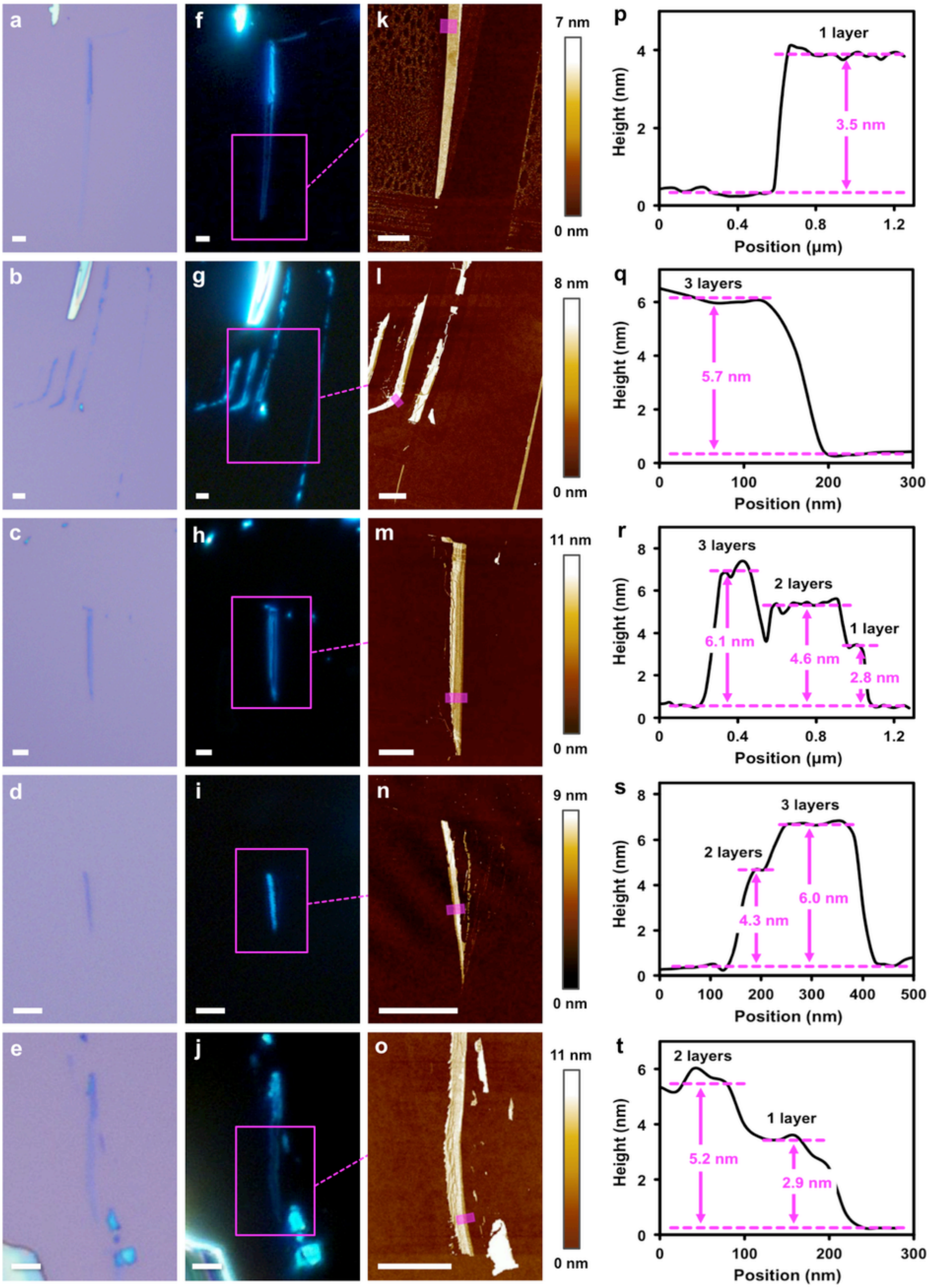

Supplementary Figure 7 | Optical and AFM characterisation of thin franckeite crystals. **a**–**e,** BF optical images of thin franckeite crystals exfoliated onto an SiO_2/Si substrate. **f**–**j,** corresponding dark-field (DF) optical images. **k**–**o,** AFM images of a selected area indicated by magenta rectangles in (**f**–**j**). **p**–**t,** Step-height profiles taken from areas indicated by the transparent magenta rectangles in the (**k**–**o**). All scale bars corresponds to 2 μm.

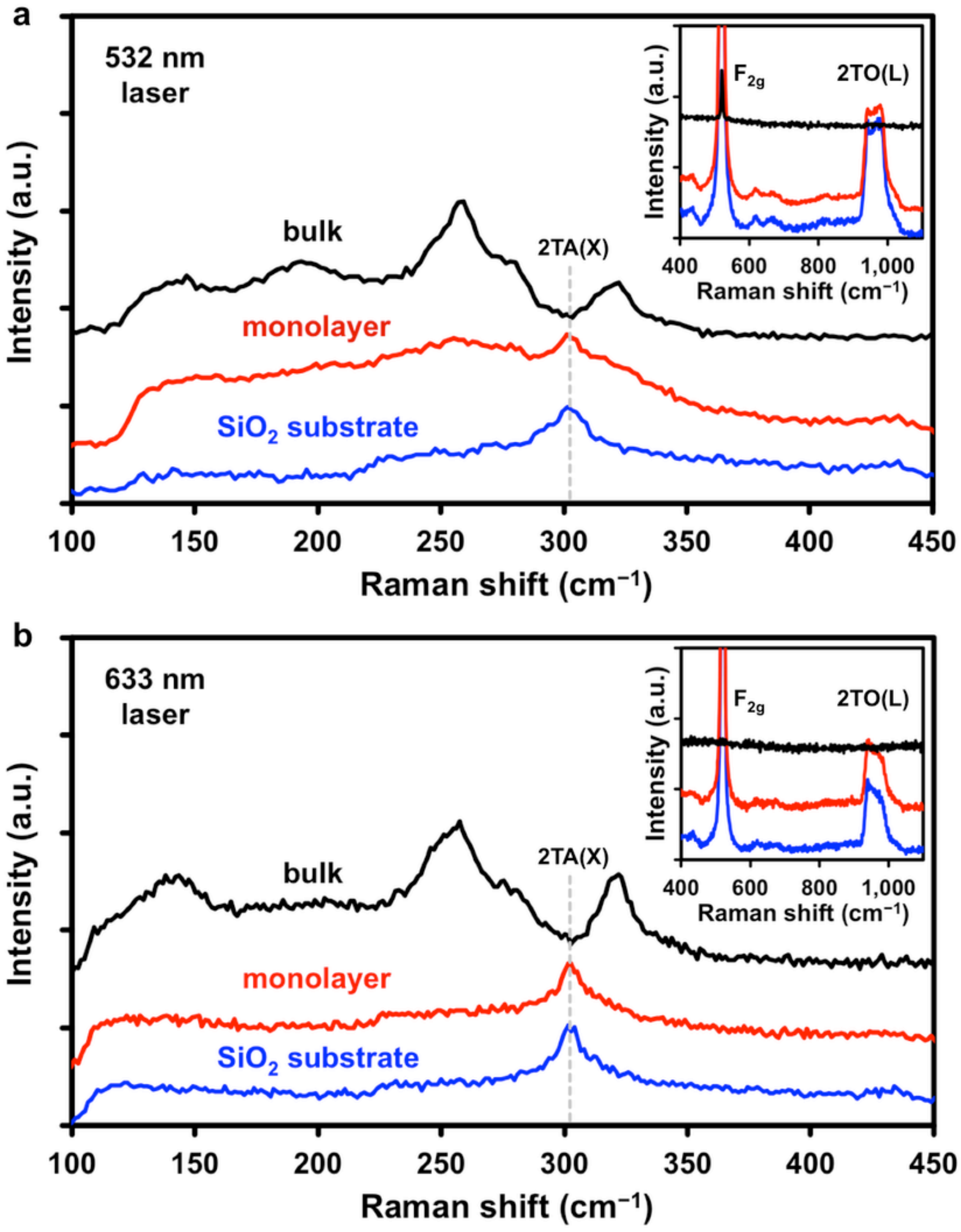

Supplementary Figure 8 | Raman spectra of monolayer franckeite, bulk franckeite, and SiO_2/Si substrate. a–b, Raman spectra of monolayer franckeite, bulk franckeite, and the SiO_2/Si substrate, using a 532 nm laser at 19 kW cm^{-2} power density (**a**) and 633 nm laser at 14 kW cm^{-2} power density (**b**). The insets show the spectral region encompassing the first- and second-order Raman Si bands. The spectra are vertically offset for clarity. More than 130 individual measurements were collected at different locations to accumulate these spectra.

.

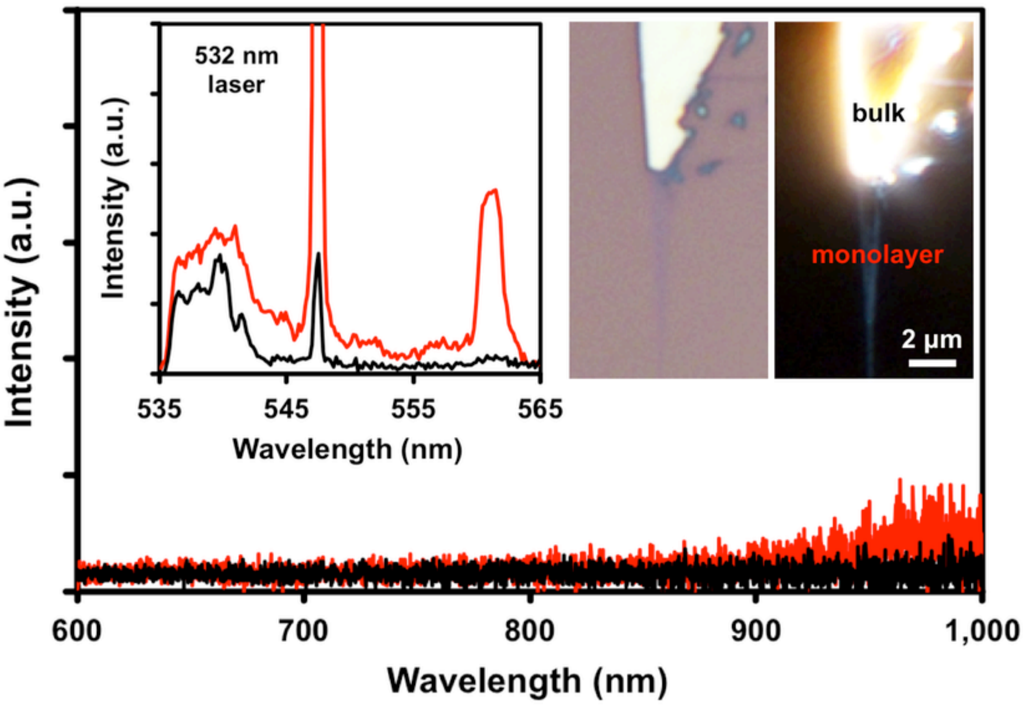

Supplementary Figure 9 | PL measurement of monolayer and bulk franckeite. Representative PL spectra of a monolayer (red) and bulk (black) franckeite crystal using 532 nm laser excitation wavelength. No PL was observed within the range of 532 – 900 nm. The inset on the left shows the Raman spectra on the same intensity scale. The insets on the right are BF and DF optical images of the measured crystals. The spectra were recorded at 35 kW cm^{-2} laser power density for 30 s.

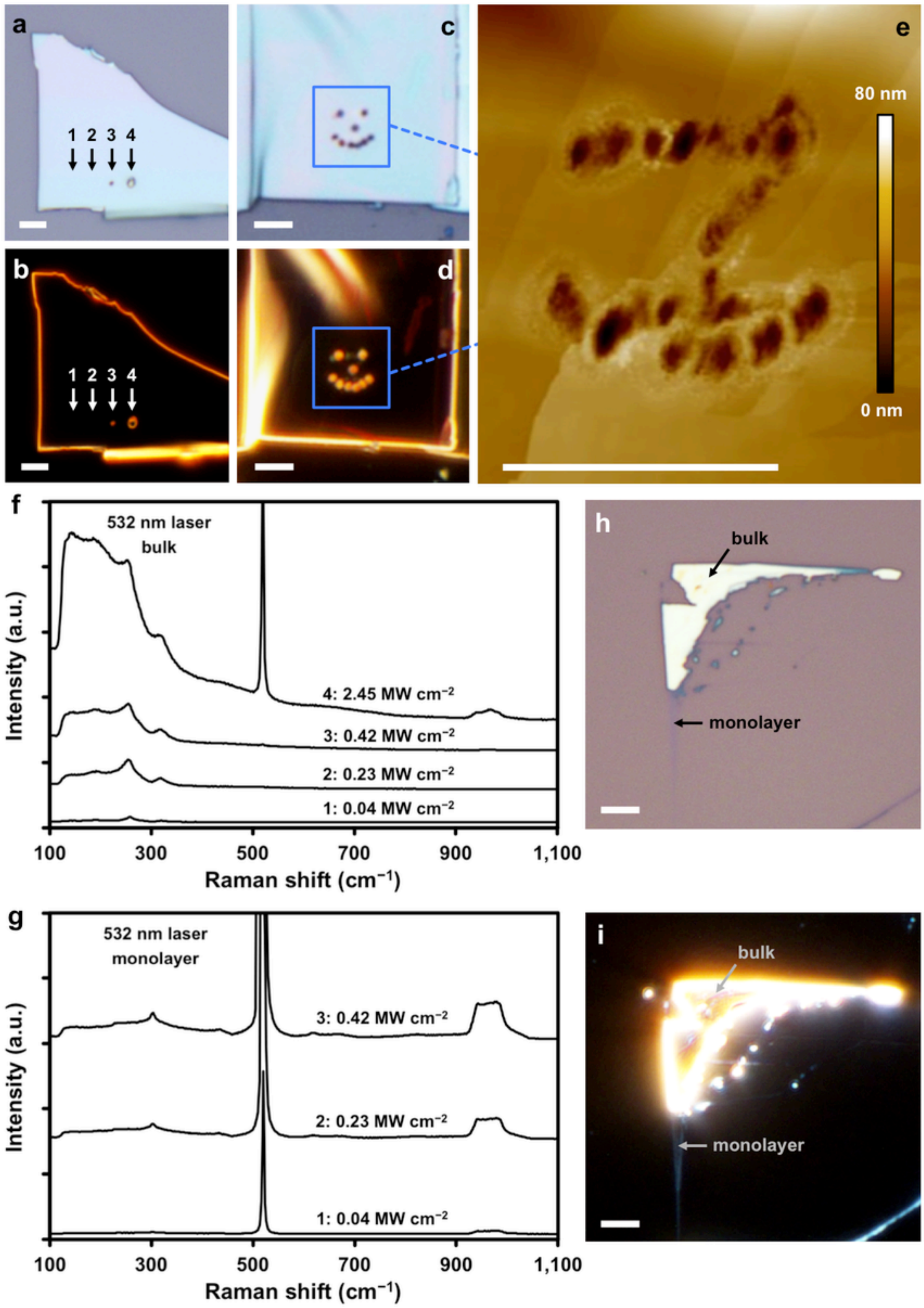

Supplementary Figure 10 | Laser-induced degradation of franckeite. a–b, BF and DF optical images of bulk franckeite subjected to increasingly higher laser irradiance for 30 s (1, 2, 3, and 4 correspond to 0.04, 0.23, 0.42, and 2.45 MW cm^{-2}, respectively). **c–d**, BF and DF optical images of a laser-induced pattern using 0.42 MW cm^{-2} for 30 s per laser spot. **e**, AFM image of the same pattern. **f–g**, Raman spectra of bulk and monolayer at varied laser power density. **h–i**, BF and DF optical images of the corresponding bulk and monolayer crystals. 532 nm laser excitation wavelength was used throughout. All scale bars denote 3 μm.

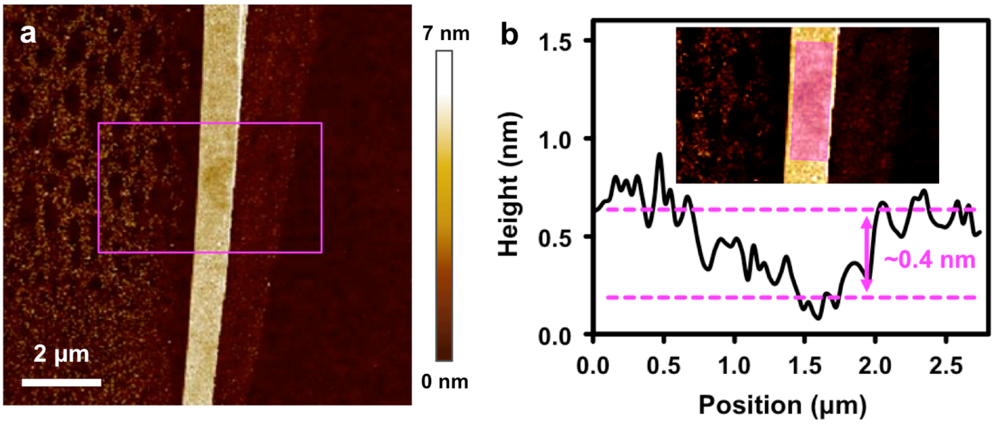

Supplementary Figure 11 | Laser ablation of franckeite surface. **a**, AFM image of a monolayer franckeite flake showing a laser-induced ablation spot after 60 s exposure to 532 nm laser at 22 kW cm^{-2} power density. **b,** Step-height profile of ablation spot taken from the area indicated by a transparent magenta rectangles in the inset AFM image.

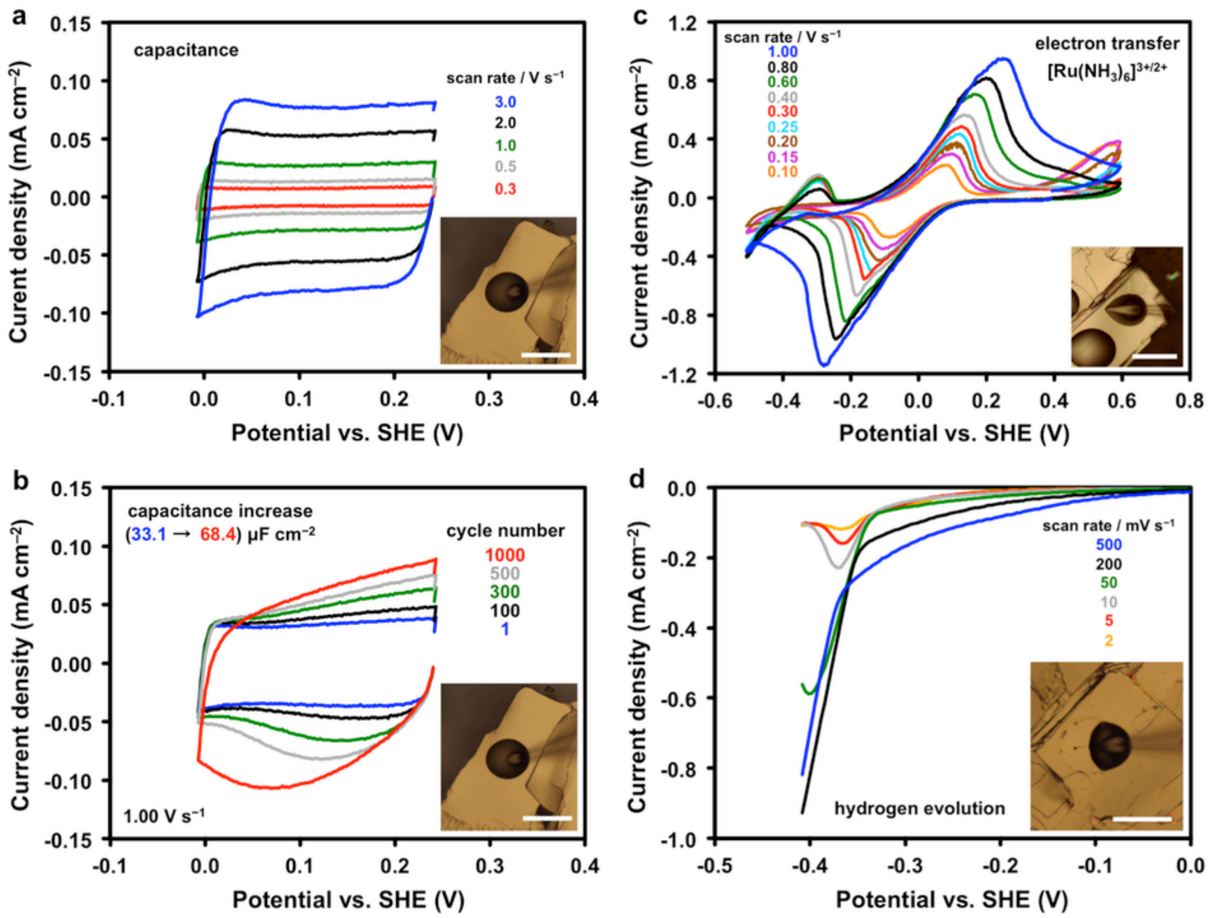

Supplementary Figure 12 | Capacitance, electron transfer, and hydrogen evolution measurements. a, Capacitance measurement using cyclic voltammetry at varied scan rates (6 M LiCl). **b**, Capacitance dependence on consecutive voltammetric cycling. **c**, Electron transfer measurement using cyclic voltammetry at varied scan rate (with a $[Ru(NH_3)_6]^{3+/2+}$ redox mediator in 6 M LiCl). **d**, Hydrogen evolution measured using linear sweep voltammetry at varied scan rates (1 M HCl in 6 M LiCl). Optical images of the micro-droplet cells used for the measurement are shown in the insets. All scale bars denote 50 μm.

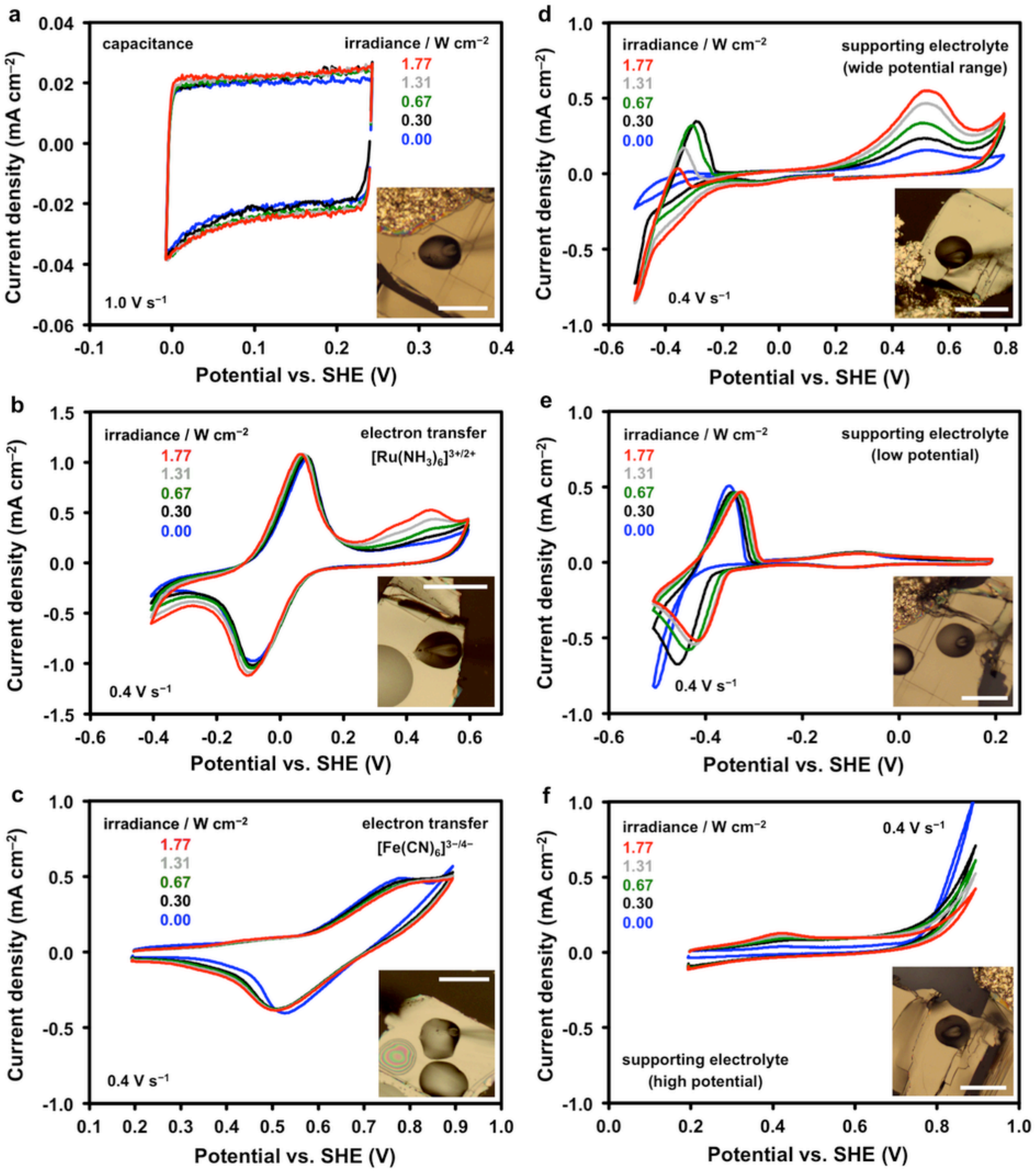

Supplementary Figure 13 | Voltammetry under varied illumination intensity. a−c, Measurements of capacitance in 6 M LiCl, electron transfer using $[Ru(NH_3)_6]^{3+/2+}$, and electron transfer $[Fe(CN)_6]^{3-/4-}$ at varied irradiance (illumination intensity). **d−f**, Wide range, low, and high potential voltammograms in 6 M LiCl supporting electrolyte at varied irradiance. Optical images of the liquid micro-droplets on franckeite surface, which were used for the measurement, are shown in the insets. All scale bars denote 50 μm.

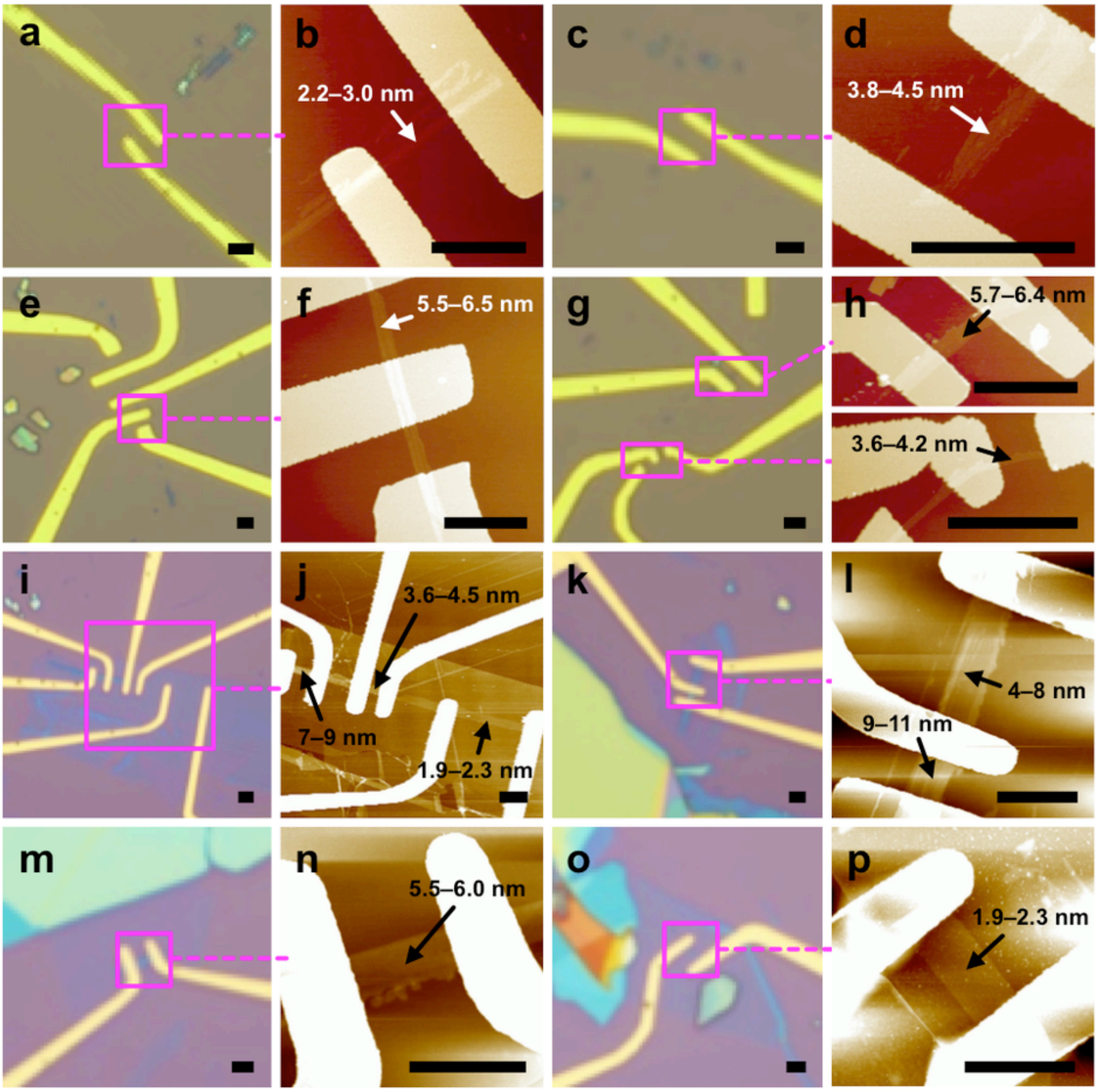

Supplementary Figure 14 | Device fabrication and characterisation. a,c,e,g,i,k,m,o, BF optical images of franckeite devices fabricated for transport and EFM measurements. **b,d,f,h,j,l,n,p,** Corresponding AFM images taken from areas indicated by the magenta rectangles showing the average flake thickness for individual channels. Devices in **a**−**h** have been prepared by direct exfoliation of franckeite onto an SiO_2/Si substrate, devices in **i**−**p** have additionally been encapsulated in a protective layer of hBN. All scale bars corresponds to 2 μm.

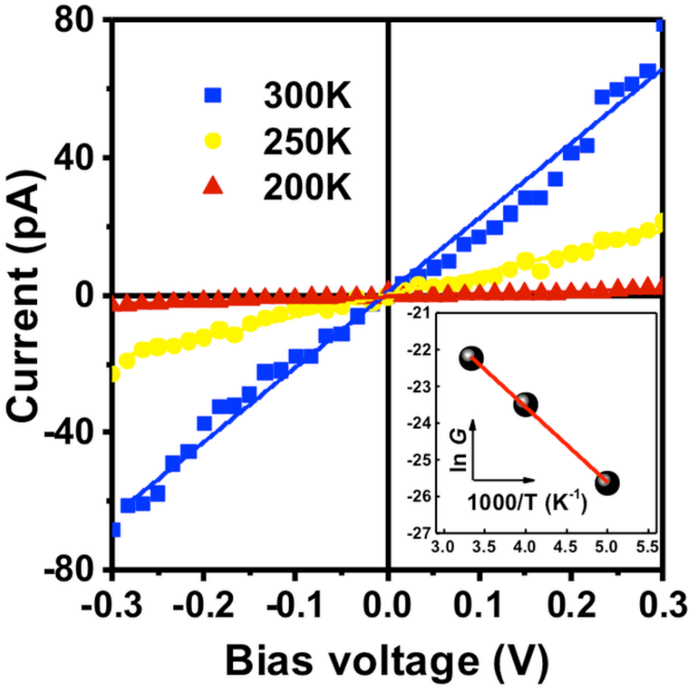

Supplementary Figure 15 | Transport characterisation of a 4-layer franckeite crystal. Current-bias voltage measurement as a function of temperature for a 4-layer franckeite device. Inset shows the dependence of the zero-bias conductance (*G*) on temperature, the solid red line is the Arrhenius fit.

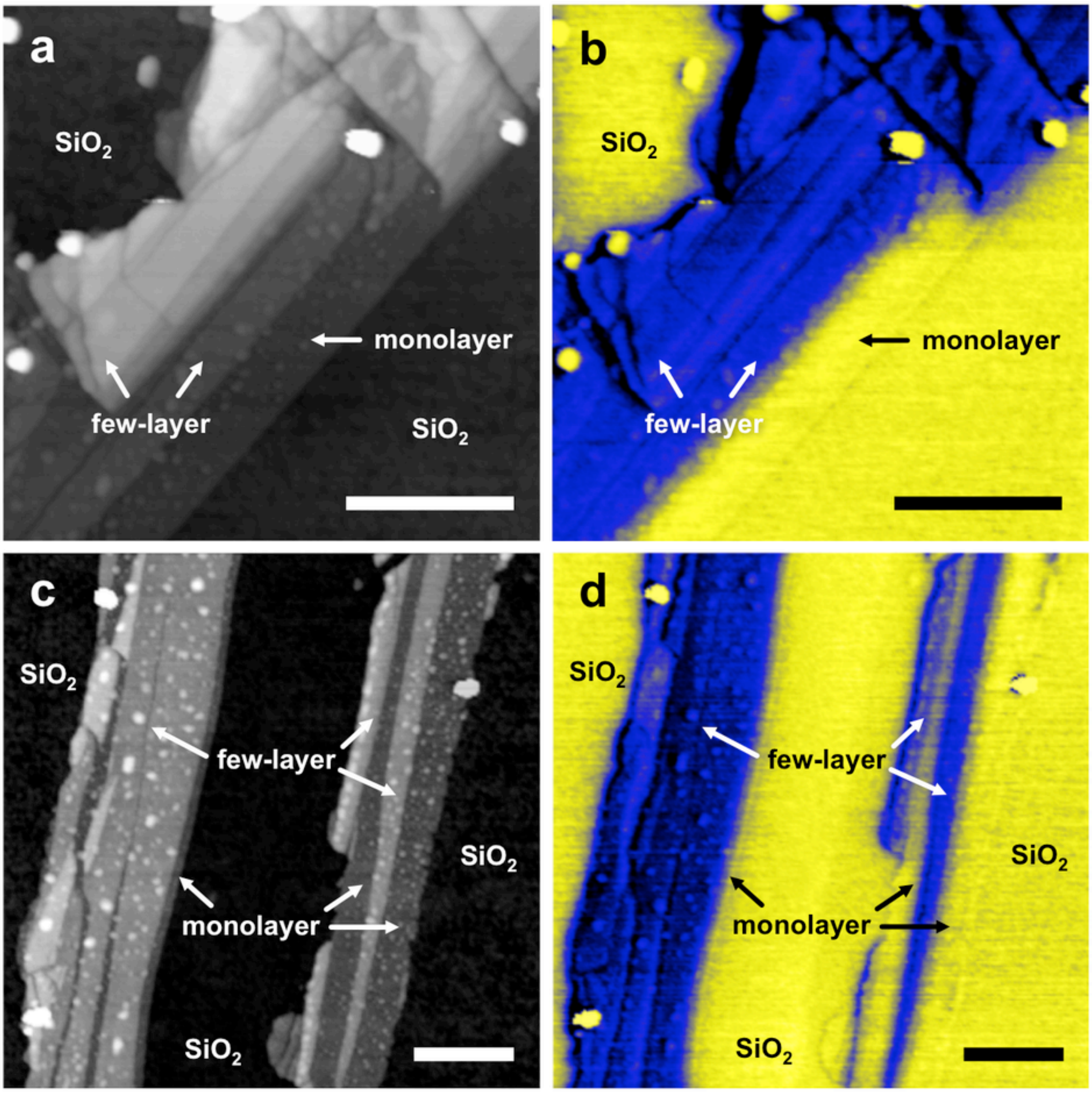

Supplementary Figure 16 | Electrostatic force microscopy measurements. a,c, Topography (first-pass) images of franckeite monolayer and few-layer regions. **b,d,** Corresponding EFM-phase (second-pass) images with +3 V DC voltage applied to the tip. The conducting and insulating regions are imaged in blue and yellow hues, respectively. All scale bars correspond to 500 nm.

Supplementary Table 1 | EDXS quantification of bulk franckeite including impurities.

element	quantity / at%
Pb	21.1 ± 4.2
Sn	10.9 ± 2.2
Sb	8.9 ± 1.8
Fe	4.0 ± 0.3
S	42.5 ± 2.7
O*	3.7 ± 0.5
C*	7.7 ± 0.9
Ag*	1.1 ± 0.3

*surface contamination and bulk impurities

Supplementary Table 2 | Composition stoichiometry parameters determined from XPS.

species	x_i	f_j	z_j
Pb^{2+}	0.238	0.815	2
Pb^{4+}		0.185	4
Sn^{2+}	0.078	0.785	2
Sn^{4+}		0.215	4
Sb^{3+}	0.097	0.637	3
Sb^{5+}		0.363	5
O^{2-}	0.038	0.243*	−2
$O_{(carb.)}$		0.757	N/A
S^{2-}	0.549	1.000	−2

*oxide fraction determined from the charge neutrality condition, Supplementary Equation (3)

Supplementary Table 3 | Elemental stoichiometric coefficients.

element	v_i	$4.2 \times v_i$	$25.2 \times v_i$
Pb	0.24	1.00	6.00
Sn	0.08	0.33	1.97
Sb	0.10	0.41	2.44
O	0.04	0.16	0.96
S	0.55	2.31	13.83

Supplementary Note 1

Scanning/transmission electron microscopy and energy-dispersive X-ray spectroscopy. Additional scanning electron microscopy (SEM) images of franckeite crystals at four different magnifications are shown in Supplementary Figures 1a–d. Low-magnification image (Supplementary Figure 1a) shows the ‘crumbly’ character of these crystals at the millimetre scale. High-magnification images (Supplementary Figures 1b–d) reveal a layered nature of franckeite at the micrometre scale, which facilitates mechanical exfoliation to thin, needle-like crystals, thus yielding a large surface area material, suitable for applications in energy storage, conversion, and catalysis. Transmission electron microscope (TEM) energy-dispersive X-ray spectroscopy (EDXS) mapping of the elemental distribution within a franckeite crystal is shown in Supplementary Figures 1e–l. The EDXS maps reveal that Pb, Sn, Sb, Fe, and S (Supplementary Figures 1e–i), all display homogeneous spatial distribution, and closely follow the topography of the crystal observed in the bright-field (BF)-TEM image (Supplementary Figure 1l). This implies that all the metals and sulphur are evenly distributed throughout the material. On the other hand, this distribution-topography correlation is completely absent for carbon (Supplementary Figure 1j), which suggests that it is purely a surface contaminant. Oxygen shows somewhat weak distribution-topography correlation (Supplementary Figure 1k), which suggests that it is bound both in metal oxides and in surface carbonaceous contamination. This is also evidenced by the X-ray photoelectron spectroscopy (XPS) results – see Supplementary Note 3.

Supplementary Table 1 shows the average elemental composition determined from the EDXS spectrum of the entire crystal in Supplementary Figures 1e–l, including the main impurity elements (C, O, and Ag). Carbon concentration (~8 at%) is much lower than that determined from the XPS (~50 at%, Supplementary Figure 4), confirming that carbon is present purely as a surface contaminant. On the other hand, the relative silver concentration is similar for both techniques, suggesting that it is a bulk contaminant. As mentioned in the main text, the EDXS quantification is approximate, as is the chemical formula derived from it. The accuracy of quantification in EDXS is generally known to be no better than ~5 at% due to adsorption and geometric considerations, especially in the absence of reference standards.[2] Quantification is also notoriously challenging for the low atomic number elements

due to the effects of absorption in the sample and in the detector, so we would expect the sulphur concentration to be underestimated and its quantification errors to be largest. This is likely to be the cause for the reduction of the S content in the EDXS quantification in comparison to the expected stoichiometry for the crystal. The quantification is further complicated by the presence of impurities mentioned above (O, C, and Ag). These elements combined make up around 13 at% of the mineral (Supplementary Table 1) and they have not been taken into account in determining the literature formula of franckeite.[3]

Supplementary Note 2

Transmission/scanning transmission electron microscopy. Supplementary Figure 2 shows the indexed electron diffraction pattern and the high-resolution TEM/scanning electron microscopy (STEM) images along the [001] direction, which reveal distinct moiré fringes originating from the H (Sn-rich) and T (Pb-rich) layer lattice mismatch, discussed in the main text. We also note that it was challenging to isolate a suitable franckeite single crystal for electron diffraction,[4] and the pattern in Supplementary Figure 2a contains contributions from more than one crystal.

The sample for cross-sectional characterisation was prepared using focused ion beam (FIB) milling as shown schematically in Supplementary Figure 3a. An ex-situ sputter coating of a 7 nm thick amorphous carbon film was followed by deposition of a 2 nm thick Au-Pd film (Q150T, Quorum Technologies Ltd, Lewes, UK). A dual-beam FIB (Nova NanoLab, FEI) with FEG-SEM and gallium FIB columns were then used for a cross-section preparation with an in-situ lift out approach. The region of interest was identified using SEM and a Pt protective strip (500 nm high) was formed using electron beam-induced deposition of trimethyl (methylcyclopentadienyl) platinum(IV), followed by a FIB Pt deposition (1.5 μm high). Trenches were milled (30 kV, 10 – 1 nA) until the lamella thickness was 1 – 1.5 μm. Then, an in-situ lift out was performed using a micromanipulator (Omniprobe™), attached to the protective Pt layer of the cross-section, with further Pt deposition. The lamella was then transferred to a three-post FIB grid and thinned (30 kV at 0.5 – 0.1 nA, 5 kV at 50 pA and finally 2 kV at 90 pA) until electron transparent (20 – 50 nm in thickness).[5] The moiré pattern observed along the [001] direction (Supplementary Figures 2d and 2e) is rationalised by the

high-angle annular dark-field (HAADF)-STEM imaging in Supplementary Figure 3b, which reveals incommensurate stacking of the H (Sn-rich) and T (Pb-rich) layers in respect to one another. The crystal unit cells of these two layers along the [010] direction have different dimensions and only come into phase every ~4.4 nm (giving rise to the moiré fringes shown in Supplementary Figures 2d and 2e). Supplementary Figure 3c shows the relative displacement of the H layers and Supplementary Figure 3d shows substitution of the H layers with a continuous PbS layer under the electron beam. Supplementary Figure 3e shows the EDXS concentration profile for a 35 nm thick cross-section. Note that while the periodicity in concentration is very obvious for the four metallic elements, it is not so for sulphur, due to its low atomic number and hence lower EDXS sensitivity.

Supplementary Note 3

X-ray photoelectron spectroscopy. The compositional stoichiometry of franckeite surface based on the XPS measurements was determined using Supplementary Equations (1–4). This analysis is based on the balance between the atomic fractions and oxidation states of lead, tin, antimony, sulphur, and oxygen. Supplementary Equation (1) expresses the material balance for different elements on franckeite surface.

$$\sum_i x_i = 1 \qquad (1)$$

x_i is the atomic fraction of an element i (e.g. Pb or Sn) determined from XPS quantification. Supplementary Equation (2) expresses the material balance for different species of the same element.

$$\sum_j f_j = 1 \qquad (2)$$

f_j is the fraction of a species j (e.g. Pb^{2+} or Pb^{4+}) determined from the high-resolution XPS spectra. Supplementary Equation (3) expresses the charge neutrality condition for the chemical formula.

$$\sum_i \sum_j x_i f_j z_j = 0 \qquad (3)$$

z_j is the charge number of a species j (e.g. $z_j = -2$ for $j = S^{2-}$). The atomic fractions, species fractions, and charge numbers are listed in Supplementary Table 2. The stoichiometric coefficient v_i of an element i in the surface chemical formula is determined from Supplementary Equation (4).

$$v_i = \frac{x_i}{\sum_i x_i} = x_i \qquad (4)$$

The normalised stoichiometric coefficients of the elements are listed in Supplementary Table 3. The chemical formula was therefore determined as $Pb_{6.0}Sn_{2.0}Sb_{2.4}S_{13.8}O_{1.0}$.

The charge neutrality condition, Supplementary Equation (3), has been found to be fully satisfied assuming only ~24 % of the oxygen is bound in metal oxides. From this follows that the remaining ~76 % of oxygen is bound as surface carbonaceous adsorbates, which is also indirectly evidenced by shape of the O 1*s* peak at 532.3 eV (Fig. 3d). A large width of this peak suggests that it is, in fact, made up of several oxygen-bound species with differing binding energies (metal-oxides: ~529 – 531 eV, organic oxygen: ~531 – 533 eV).[6]

Considering previous reports of oxidation and carbonaceous contamination of other layered materials,[7, 8, 9, 10] it is useful to compare the surface composition of crystals that have been exposed to air for more than 24 h (aged surface) with those exposed immediately prior to the transfer to the XPS vacuum chamber (freshly cleaved surface). The XPS spectra obtained on aged and freshly cleaved franckeite surface are shown in Supplementary Figures 4a and 4b, respectively. Quantification tables embedded in Supplementary Figures 4a and 4b suggest that the variations in element quantities are similar for both aged and freshly cleaved surfaces. There is a small increase in the average amount of surface carbon (by 13%) and oxygen (by 20%). Overall, it can be concluded that the differences between the aged and freshly cleaved surface are small and one would have to significantly decrease the time between the cleaving and XPS measurement in order to observe more pronounced changes. Adventitious carbon and trace elements (Ag, Fe, Cl, and F, all below 1 at%), which were observed intermittently, were not included in this analysis. It is reasonable to assume that the amounts of positively and negatively charged species of these elements will more or less balance out, thus contributing a near-zero net charge to the chemical formula, having little

impact on the stoichiometric analysis described above. The differences between the EDXS and XPS quantification, in particular the relative concentrations of Sn and Fe, which are related to the difference between bulk and surface of franckeite crystals, are discussed in the main text.

Supplementary Figure 5 displays a high-resolution XPS spectra of the minority metallic elements, silver and iron, and the adventitious surface carbon. Supplementary Figure 5a shows the Ag 3*d* doublet peak. Deconvolution of the Fe 2*p* doublet peak, superimposed on the Sn $3p_{3/2}$ peak, is shown in Supplementary Figure 5b. Note that silver, as well as iron, are common contaminants in galenite (PbS) and are therefore likely to follow more complex galenite-derived minerals, such as franckeite.[3, 11] The C 1*s* peak and its deconvolution to three components are shown in Supplementary Figure 5c. Due to the large variation in the reported values of organic carbon binding energies and for the sake of simplicity, we have grouped the carbon-bound species into three categories, based on their approximate binding energy.[6] These are: 1) carbon-carbon sp^2 and sp^3 bonds (red), 2) carbon-hydrogen and carbon-oxygen (single) bonds (blue), and 3) carbon-oxygen (double) and carbon-halogen bonds (green).

Supplementary Note 4

Optical microscopy, Raman spectroscopy, and atomic force microscopy. Occasionally, terraces of franckeite with sub-monolayer thickness (< 1.85 nm) were observed. Supplementary Figure 6 shows atomic force microscopy (AFM) characterisation of such case, for a thin franckeite flake encapsulated in a hexagonal boron nitride (hBN) crystal. Thickness of the sub-monolayer terraces, which are not visible under an optical microscope, varies between 0.7 – 1.5 nm (height profiles are shown in Supplementary Figures 6b–c). This could either be a result of local faults in the crystal structure and/or an indirect evidence that the exfoliation of franckeite can also occur at other than the van der Waals planes, e.g. half way through the T layer. This scenario is also supported by the TEM results (Supplementary Figure 3d and Fig. 2e). As mentioned in the main text, the length of the exfoliated few-layer crystals was typically on the order of tens of μm, but their width rarely exceeded 0.5 – 1 μm, resulting in characteristic needle-like crystals with a high length-to-width aspect ratio. We have exfoliated a large number of thin franckeite crystals and concluded that this is a

universal behaviour of franckeite, irrespective of the mechanical exfoliation conditions. Supplementary Figure 7 shows optical and AFM characterisation of several such crystals, which were used for the Raman spectroscopy measurements.

Supplementary Figure 8 shows the raw Raman spectra of monolayer and bulk franckeite (not corrected for the Si/SiO_2 substrate background) as well as the reference spectra of the underlying SiO_2/Si substrate for 532 nm (Supplementary Figure 8a) and 633 nm (Supplementary Figure 8b) laser excitation wavelengths. The 532 nm laser was used in most of our measurements due to the stronger Raman signal. Supplementary Figure 8 shows that the decrease in the flake thickness is accompanied by an appearance of a minor Si peak, 2TA(X) peak at 302 cm^{-1}, and two major Si peaks, F_{2g} at 520 cm^{-1} and 2TO(L) at 970 cm^{-1}, in the monolayer Raman spectra.[12] The intensities of these peaks, originating from the underlying Si, are greatly reduced in thick bulk crystals (> 100 nm thickness). There is currently no reliable Raman literature on franckeite and available databases only provide spectra recorded at high laser power densities,[13] which damage metal chalcogenides minerals.

We have also attempted to measured photoluminescence (PL) spectra of franckeite. No appreciable PL was detected within the 532 – 900 nm wavelength range from bulk or monolayer franckeite (Supplementary Figure 9), which is to be expected given the small infrared band gap determined from the transport measurements (80 – 220 meV, attributed to Schottky barrier) and predicted from theory (~350 meV). Raman spectra on the same intensity scale collected during the PL acquisition and the optical images of the measured bulk and monolayer crystals are shown in the inset of the figure.

All the Raman spectra shown so far (both here and in the main article) were recorded at low laser power density, namely ≤35 kW cm^{-2} for the green laser and ≤73 kW cm^{-2} for the red laser, and for short exposure time, typically 20 – 30 s. Franckeite crystals were also subjected to increasingly higher laser power densities of 0.04 MW cm^{-2}, 0.23 MW cm^{-2}, 0.42 MW cm^{-2}, and 2.45 MW cm^{-2}, as shown by the BF and DF optical images in Supplementary Figures 10a−b. Laser-induced degradation of the crystals is visible for the high laser power densities (≥0.42 MW cm^{-2}). Supplementary Figures 10c and 10d show BF and DF optical images of a laser-irradiation pattern obtained at 0.42 MW cm^{-2}. An AFM image of this pattern in Supplementary Figure 10e reveals radial damage, matching the dimensions of the

spherical laser spot size (~0.8 μm^2). Comparison between the laser-induced degradation of bulk and monolayer crystals was also examined. Raman spectrum of bulk crystal changes the shape at high laser power densities and is followed by an appearance of the underlying Si Raman peaks at 520 cm^{-1} and 970 cm^{-1} (Supplementary Figure 10f). Raman spectrum of the monolayer crystal shows almost complete disappearance of franckeite signal at high laser power density and an increase in the intensity of the minor Si peak at 302 cm^{-1} (Supplementary Figure 10g). The BF and DF optical images of bulk and monolayer crystals, which were used to obtain these Raman spectra are shown in Supplementary Figures 10h and 10i.

Prolonged exposure (>60 s) of monolayer franckeite to laser irradiation at low power density (22 kW cm^{-2}) has revealed signs of shallow ablation of the surface. A circular depression is observed where the laser was focused on the surface (Supplementary Figure 11a). The AFM profile of the depression reveals reduction of the flake thickness by about 0.4 nm (Supplementary Figure 11b). There are two likely explanations for this observation. First, the terminating layer of franckeite could be undergoing heat-induced removal from the surface. The shallow nature of the ablation suggests that this would be the thin H layer rather than the four-atom thick T layer. Second, the laser irradiation could be removing the ubiquitous carbonaceous adsorbates from franckeite surface. In order to prevent even a partial degradation of franckeite, we used significantly shorter exposure time (20 s) for the collection of the Raman spectra in the main text.

Supplementary Note 5

Electrochemical measurements. Cyclic voltammograms in a pure electrolyte (for the capacitance measurement) obtained at varied scan rates and their dependence on consecutive cycling at a constant scan rate are shown in Supplementary Figures 12a and 12b, respectively. Cyclic voltammograms with a redox mediator (for the electron transfer measurement) at varied scan rates are shown in Supplementary Figure 12c. Cyclic voltammograms in hydrochloric acid (for the hydrogen evolution measurement) are shown in Supplementary Figure 12d. The basal surface of franckeite is very flat (root mean squared roughness, R_q, was typically 0.5 – 1.0 nm) and therefore the geometric/active surface area effects are negligible.

On the other hand, the uncertainty in the geometric vs. active surface area on edge/defective surfaces means that their electrochemical performance is more difficult to quantify.

The capacitance and electron transfer measurements were also examined at varied white light illumination intensity (irradiance), results of which are shown in Supplementary Figure 13. Illumination of the crystals was realised using a 20× optical microscope objective and the irradiance was calibrated using the 843-R power meter (Newport Spectra-Physics Ltd, UK). Supplementary Figures 13a, 13b, and 13c show the cyclic voltammograms in a pure 6 M LiCl electrolyte, in an electrolyte with a $[Ru(NH_3)_6]^{3+/2+}$ redox mediator, and in an electrolyte with a $[Fe(CN)_6]^{3-/4-}$ redox mediator, respectively, obtained at constant scan rate and varied irradiance. Only small changes are observed with varying irradiance and they are likely to be irradiation-induced thermal effects. This agrees with our transport measurements of franckeite's narrow infrared band gap (0.1 – 0.2 eV). The capacitance calculated from equation (3) slightly increases (by ~15 %) for the maximum irradiance in comparison to the zero-irradiance. The peak-to-peak separation of the $[Ru(NH_3)_6]^{3+/2+}$ reduction/oxidation decreases by 8 mV for the maximum irradiance, indicating a slightly faster electron transfer (by ~14 %) in comparison to the zero-irradiance. Dependence of the $[Fe(CN)_6]^{3-/4-}$ reduction/oxidation peak-to-peak separation on irradiance is complex, initially increasing and then decreasing slightly at high irradiance. It is possible that the $[Fe(CN)_6]^{3-/4-}$ mediator adsorbs at the surface and obscures the measurement, as observed previously on graphite.[7] In a word, small changes in the capacitance and electron transfer rate indicate franckeite is a degenerate, narrow band gap semiconductor.

The voltammetric background in pure supporting electrolyte over a large potential range was also examined at varied irradiance. Series of cyclic voltammograms in Supplementary Figure 13d show a redox activity in a wide potential region, which significantly increases with increasing irradiance. In order to deconvolute the contribution of the low and high potentials to the overall activity, separate voltammograms of the low and high potential regions were recorded (Supplementary Figures 13e and 13f). Analysis of the voltammograms indicates that the broad oxidative peak, centred around +0.5 V is associated with the reductive current at low potentials (below −0.35 V). Performing voltammetry in the low potential region (Supplementary Figure 13e) reveals a well-defined redox process, which

becomes near-reversible at highest irradiance. When voltammetry is performed within the high potential region (Supplementary Figure 13f) the peak at +0.5 V disappears and the potential window is extended to ca. +0.8 V. This redox activity most likely contributes to the increased inherent capacitance within the narrow potential window (0 – 0.25 V vs. SHE) and therefore results in a pseudo-capacitive behaviour. The redox activity undoubtedly originates from transitions between different oxidation states of the metals, although the exact peak assignment is difficult due to uncertainties in metal sulphides' standard redox potentials.[14]

Supplementary Note 6

Transport and electrostatic force microscopy measurements. A large number of devices have been fabricated for the transport and electrostatic force microscopy (EFM) characterisation. Supplementary Figure 14 shows a selection of thin franckeite flakes ranging from monolayer (~2 – 3 nm) to 5 layers (~10 nm), electrically connected using a deposition of Cr/Au contacts. The devices shown in Supplementary Figures 14a−h have been prepared by direct exfoliation of franckeite onto an SiO_2/Si substrate, while the devices in Supplementary Figures 14i−p have additionally been encapsulated in a thin layer of hBN in order to protect franckeite crystals from oxidation and contamination.

Supplementary Figure 15 shows additional transport measurement results for a 4-layer thick franckeite crystal. The current-bias voltage curves were measured at three different temperatures, and from the zero-bias conductance on temperature in the inset, the Arrhenius fit was plotted and activation energy of 170 meV extracted.

EFM phase measurements in a double-pass mode were carried out to investigate the conductivity of thin franckeite flakes. In the first-pass, topography of the layers was imaged in a non-contact AFM mode. In the second-pass taken along the same scan line, the tip was lifted to 30 nm height from the surface while mechanically oscillating on resonance (out-of-feedback) and a direct current (DC) bias voltage was applied between the doped silicon tip (Nanosensors PPP-FMR, 0.5 – 9.5 N/m) and the underlying doped silicon substrate. The second-pass phase images directly map the conductivity of the sample by probing the second derivative of the tip-substrate capacitance, d^2C/dz^2, showing negative contrast (larger

capacitance) for conductive nanostructures over an insulating substrate and no or positive contrast (smaller capacitance) for insulating nanostructures as previously described.[15, 16] Supplementary Figure 16 shows the AFM topography images and the corresponding EFM phase images recorded using a +3V DC bias voltage. Blue hues in Supplementary Figures 16b and 16d indicate conductive material and correspond to bilayer and thicker franckeite. On the other hand, yellow hues indicate insulating material and correspond to the SiO_2 substrate and monolayer franckeite.

Supplementary Note 7

Density functional theory calculations of the electronic band structure. The optimal crystal structure of T and H layers have been calculated within the local density approximation (LDA) of density functional theory using the VASP code.[17] A plane-wave cut-off of 400 eV and a 12 × 12 **k-**point grid was used in the calculations. The structure was approximated so that no Sn and Fe atoms were assumed to be present in the T and H layer, respectively. This corresponds to approximating the T layer with Pb_3SbS_4 stoichiometry and the H layer with SnS_2 stoichiometry, based on the EDXS concentration profiles. The lattice parameters of the T layer are a = 5.735 Å and b = 5.730 Å, and the lattice parameters of the H layer are a = 3.616 Å and b = 6.264 Å. These compare well with the previously reported experimental values obtained for bulk franckeite,[18] which found a = 5.805(8) Å and b = 5.856(16) Å for the T layer, and a = 3.665(8) Å and b = 6.2575(16) Å for the H layer.

The LDA was utilized to calculate the band structure of the T and H layers and the T−H heterostructure, i.e. franckeite monolayer (Figure 7). The results show that the T layer exhibits a small direct band gap of 0.36 eV at the C point, while the H layer has a large indirect band gap of 1.48 eV, with the conduction band minimum at the C point and the valence band maximum half way between the Y and Γ points. A scissor correction of these band gaps can be performed by calculating the density of states (DOS) using the HSE06 hybrid density functional, which yields corrected band gaps of 0.79 eV and 2.46 eV for the T and H layers, respectively.

Experiments indicate that the in-plane lattice vectors of the T and H layers are aligned in bulk franckeite, hence it is in principle possible to construct a super-cell geometry out of the T and H layers to study the T−H heterostructure (franckeite monolayer). The mismatch between the lattice parameters requires a relatively large super-cell, and even then, each lattice must undergo some stretching or compression. A compromise can be achieved by setting a tolerance of 0.5 Å as the mismatch between the lattice parameters of the stretched layer super-cells and their non-stretched counterparts; in this case it is sufficient to use a single unit cell along the **b** lattice vector, while along the **a** vector 5 cells of the T layer unit cell and 8 cells of the H layer unit cell must be taken. The resulting T–H heterostructure contains 128 atoms with a = 28.8 Å and b = 5.996 Å. The distance between the mean planes of the T and H layer was optimized and found to be 9.054 Å. Note that this is slightly larger than the separation in bulk franckeite, for which the optimal distance was found to be 8.894 Å. Note also, that in the bulk calculation the approximation was made so that the **c** vector is perpendicular to the plane stretched by the **a** and **b** vectors. T−H heterostructure (monolayer) band structure displays a prominent energy gap just below the Fermi level. This gap is indirect with the conduction band minimum at the Γ point and the valence band maximum at the C point, and its magnitude is 0.35 eV, which is almost identical with that of the T layer (0.36 eV). The finding that the T−H heterostructure is metallic is a consequence of the alignment of the bands in the T and H layers seen in Figure 7. The T layer dopes the H layer with electrons due to the relative position of the Fermi levels, hence the T−H heterostructure is a type III broken-gap semiconductor interface. The number of electrons transferred corresponds to one electron per Pb_3SbS_4 unit of the T layer. The appearance of an energy gap below the Fermi level is in agreement with this finding as well.

The DOS obtained for this super-cell T–H heterostructure (monolayer) predicts that it is metallic (Figure 7d). A similar finding can be seen for the T–H–T heterostructure (one and a half monolayer), the T–H–T–H heterostructure (bilayer), and bulk franckeite. It is worth noting that most of the major DOS features in these heterostructures are the same, except for the size of the energy gap below the Fermi level, which decreases with increasing number of layers.

The situation is expected to be quite different if the non-zero concentration of Sn in the T layer and Fe in the H layer were to be taken into account. The XPS spectra indicate that franckeite contains both 2+ and 4+ valence states of Sn. Assuming Sn^{4+} cations are present in the H layer, their substitution by Fe^{2+} cations will introduce acceptor states within the H layer. Similarly, substitution of Sb^{3+} cations within the T layer by Sn^{4+} cations will create donor states within the T layer, as will substitution of Pb^{2+} cations by Sb^{3+} cations, which is an important substitution to consider given the approximation made in the modelling of the T layer. As an example, let us consider a slightly exaggerated T layer composed of only Pb^{2+}, Sb^{3+}, and S^{2-} ions, in a stoichiometry of $Pb_6Sb_2S_8$. Substitution of a half of the Sb^{3+} cations with Sn^{4+} cations and of one in every six Pb^{2+} ions with Sb^{3+} cations corresponds to turning $Pb_6Sb_2S_8$ into $Pb_5Sb_2SnS_8$, gaining two extra electrons from these substitutions. From the DOS, the number of electrons removed from the T layer in the T−H heterostructure is one electron per Pb_3SbS_4, which is an equivalent of two electrons per $Pb_6Sb_2S_8$ unit. The above substitution counteracts the removal of electrons and promotes the formation of a gapped system. It is therefore expected that the substitutions, which were neglected in the DFT calculations, will create donor states in the T layer and acceptor states in the H layer. This will drive the system towards a gapped semiconductor state due to the acceptor states countering the rise of the Fermi level in the H layer and the donor states countering the fall of the Fermi level in the T layer, which is energetically favourable. An important implication of this finding is that the T layer with the substitutions included contains free charge carriers in the conduction band, while the H layer contains charge carriers (holes) in the valence band. This therefore predicts that successful isolation of either T or H layer of franckeite would yield a 2D doped semiconductor.

Supplementary References

1. Barr T. L.& Seal S. Nature of the use of adventitious carbon as a binding energy standard. *J. Vac. Sci. Technol., A* **13**, 1239-1246 (1995).
2. Williams D. B.& Carter C. B. *Transmission Electron Microscopy: A Textbook for Materials Science*. Springer (2009).
3. Moh G. H. Mutual Pb^{2+}/Sn^{2+} substitution in sulfosalts. *Mineral. Petrol.* **36**, 191-204 (1987).
4. Wang S.& Kuo K. H. Crystal lattices and crystal chemistry of cylindrite and franckeite. *Acta Crystallogr., Sect. A: Found. Crystallogr.* **47**, 381-392 (1991).
5. Schaffer M., Schaffer B.& Ramasse Q. Sample preparation for atomic-resolution STEM at low voltages by FIB. *Ultramicroscopy* **114**, 62-71 (2012).
6. Benoit R. La Surface: XPS database; http://www.lasurface.com, accessed 30/04/2016. CNRS Orléans (2016).
7. Patel A. N.*, et al.* A new view of electrochemistry at highly oriented pyrolytic graphite. *J. Am. Chem. Soc.* **134**, 20117-20130 (2012).
8. Velický M.*, et al.* Electron Transfer Kinetics on Mono- and Multilayer Graphene. *ACS Nano* **8**, 10089-10100 (2014).
9. Velický M.*, et al.* Electron transfer kinetics on natural crystals of MoS_2 and graphite. *Phys. Chem. Chem. Phys.* **17**, 17844-17853 (2015).
10. Nioradze N., Chen R., Kurapati N., Khvataeva-Domanov A., Mabic S.& Amemiya S. Organic Contamination of Highly Oriented Pyrolytic Graphite As Studied by Scanning Electrochemical Microscopy. *Anal. Chem.* **87**, 4836-4843 (2015).
11. Young C. A., Taylor P. R.& Anderson C. G. *Hydrometallurgy 2008: Proceedings of the Sixth International Symposium*. Society for Mining, Metallurgy, and Exploration (2008).
12. Wang R.-P.*, et al.* Raman spectral study of silicon nanowires: High-order scattering and phonon confinement effects. *Phys. Rev. B: Condens. Matter Mater. Phys.* **61**, 16827-16832 (2000).
13. Downs B. RRUFF Project: Mineral characterization database, http://www.rruff.info/franckeite/, accessed 26/04/2016. Department of Geosciences, University of Arizona (2016).
14. Licht S. Aqueous Solubilities, Solubility Products and Standard Oxidation-Reduction Potentials of the Metal Sulfides. *J. Electrochem. Soc.* **135**, 2971-2975 (1988).
15. Bockrath M.*, et al.* Scanned Conductance Microscopy of Carbon Nanotubes and λ-DNA. *Nano Lett.* **2**, 187-190 (2002).
16. Datta S. S., Strachan D. R., Mele E. J.& Johnson A. T. C. Surface Potentials and Layer Charge Distributions in Few-Layer Graphene Films. *Nano Lett.* **9**, 7-11 (2009).
17. Kresse G.& Furthmüller J. Efficient iterative schemes for *ab initio* total-energy calculations using a plane-wave basis set. *Phys. Rev. B: Condens. Matter Mater. Phys.* **54**, 11169-11186 (1996).
18. Makovicky E., Petříček V., Dušek M.& Topa D. The crystal structure of franckeite, $Pb_{21.7}Sn_{9.3}Fe_{4.0}Sb_{8.1}S_{56.9}$. *Am. Mineral.* **96**, 1686-1702 (2011).
